# Overexpression of a Vesicle Trafficking Gene, OsRab7, Enhances Salt Tolerance in Rice

**DOI:** 10.1155/2014/483526

**Published:** 2014-02-12

**Authors:** Xiaojue Peng, Xia Ding, Tianfang Chang, Zhoulong Wang, Rong Liu, Xin Zeng, Yaohui Cai, Youlin Zhu

**Affiliations:** ^1^Key Laboratory of Molecular Biology and Gene Engineering of Jiangxi Province, College of life science, Nanchang University, Nanchang 330031, China; ^2^Jiangxi Super-rice Research and Development Center, Nanchang 330200, China

## Abstract

High soils salinity is a main factor affecting agricultural production. Studying the function of salt-tolerance-related genes is essential to enhance crop tolerance to stress. *Rab7* is a small GTP-binding protein that is distributed widely among eukaryotes. Endocytic trafficking mediated by *Rab7* plays an important role in animal and yeast cells, but the current understanding of *Rab7* in plants is still very limited. Herein, we isolated a vesicle trafficking gene, *OsRab7*, from rice. Transgenic rice over-expressing *OsRab7* exhibited enhanced seedling growth and increased proline content under salt-treated conditions. Moreover, an increased number of vesicles was observed in the root tip of *OsRab7* transgenic rice. The *OsRab7* over-expression plants showed enhanced tolerance to salt stress, suggesting that vacuolar trafficking is important for salt tolerance in plants.

## 1. Introduction 

High soil salinity is a major environmental stress inhibiting plant growth and development, and it results in severe yield loss in agricultural crops. Plants exhibit various responses to increase their capability to resist salt stress. Recent studies demonstrated that proteins involved in intracellular membrane trafficking in the secretory and endocytic pathways play an important role in plant defense against pathogens and stress adaptation [1].

Vesicle trafficking has been traditionally viewed as a constitutive housekeeping process, but recent reports have described the involvement of intracellular vesicle trafficking in the resistance to environmental stresses. In *E. coli, *mutations that cause increased vesiculation enhanced bacterial survival upon challenge with stressing agents or the accumulation of toxic misfolded proteins [2]. In yeast, vesicle trafficking between the cytosoll and plasma membrane was inhibited by oxidative stress [3]. In *Arabidopsis thaliana*, the transgenic plants in which the expression of an autophagy-related gene* AtATG18a* was knocked down were more sensitive to salt and osmotic stress [4].

The Rab family is conserved from yeast to animals and has been implicated in intracellular vesicle trafficking and membrane organization [5]. In *Arabidopsis*, it is reported that the *Rab7*-related protein appears to localize to the vacuolar membrane and regulate the vesicle fusion with the vacuole [6]. In soybean, however, the presence of *Rab7*-related protein was found on both endosomes and tonoplasts, indicating that *Rab7* multivesicular bodies are participated in endocytosis pathway. In addition, many plant *Rab7* genes were shown to be affected by environmental stressors, and transgenic experiments demonstrated that the overexpression of the *AtRab7* and *PgRab7* could increase stress tolerance in transgenic *Arabidopsis thaliana* and transgenic tobacco, respectively [6, 7].

*OsRab7* transcripts were found to accumulated slightly under salt stress in rice [8], but direct evidence showing that the overexpression of *OsRab7 *can increase tolerance to salt stress has not been reported. In this paper, we prepared transgenic rice plants that overexpressed *OsRab7 *and tested their tolerance to salt stress. The transgenic plants exhibited enhanced tolerance to salt stress and increased numerous distributed vesicles in the root tips.

## 2. Materials and Methods

### 2.1. Plant Materials and Growth Conditions

The japonica rice variety Zhonghua 11 was used in this study. T_0_, T_1_, and T_2_ progeny transgenic plants were grown at 25°C under 16 h of daylight in a greenhouse. For the physiological analysis, the plants were grown under a controlled temperature of 23°C at 70% relative humidity.

### 2.2. Gene Cloning and Rice Transformation

Total RNA was isolated from rice seedlings with Trizol reagent (Invitrogen). Reverse transcription was performed using MMLV Reverse Transcriptase (Invitrogen) according to the manufacturer's directions. To obtain the full length sequence of *OsRab7,* a pair of primers was designed according to the *OsRab7* cDNA sequence. The primer sequence was forward: 5′-ATGGCTTCGCGCCGC-3′, Reverse: 5′-CTAGCAGCAGCCTGATGATCTTG-3′. The *OsRab7* cDNA was digested with BamHI and ligated to the modified binary vector plasmid pCU [9]. The resulting constructs were designated *pOsRab7*. The japonica rice variety zhonghua 11 was transformed using *Agrobacterium tumefaciens* EHA-105, following the published procedures published by Hiei et al. [10].

### 2.3. Semiquantitative RT-PCR and Real-Time RT-PCR Analysis

Total RNA was isolated and cDNA was synthesized from transgenic and wild-type rice as described above. The *actin* gene was used as an internal control for RT-PCR analysis. General PCR was performed using the following program: an initial denaturation at 94°C for 3 min, followed by 25 cycles of 94°C for 45 s, 60°C for 45 s, and 72°C for 1 min, and a final extension at 72°C for 10 min. The RT-PCR experiments were repeated three times, and the PCR products were detected in a 1% agarose gel with 1 × TAE and EtBr. Real-time PCR was performed with a Rotor-Gene 2000 real-time thermal cycling system (Corbett Research) using the SYBR Green Real-time PCR Master Mix (TOYOBO). The reaction for *OsRab7* using specific primers (sense 5′-AGCCGTGTGGTCTCTGAG-3′; antisense 5′-AATGGGTCCTTGAGAGTCAC-3′) was performed at 95°C for 10 s followed by 40 cycles of 95°C for 10 s, 55°C for 10 s, and 72°C for 10 s. The reaction for *actin* as internal standard using specific primers (sense 5′-AGCATGAAGATCAAGGTGGTC-3′; antisense 5′-GCCTTGGCAATCCACATC-3′) was performed in the same as that for *OsRab7.*

### 2.4. Preparation of Total Protein Extract From Rice

Total protein was extracted from transgenic seedling and wild-type seedlings. The tissues were ground into a fine powder in liquid nitrogen, and then 400–500 mg of tissue was extracted in 1 mL of extraction buffer (66 mM Tris-Cl, pH 6.8, 2% SDS, 2% v/v 2-mercaptoethanol) at room temperature followed by centrifugation for 20 min at 40000 ×g to remove the insoluble fraction. The proteins were precipitated at −20°C for 1 h by the addition of 1 mL of an 8 : 1 mixture of ice-cold acetone and trichloroacetic acid with 0.1% v/v 2-mercaptoethanol. The supernatant was discarded after centrifugation at 18000 ×g for 15 min at 4°C, and the pellet was washed in 1 mL of ice-cold acetone. The remaining acetone was removed by air drying at room temperature. The resulting pellet was redissolved in the protein extraction solution.

### 2.5. Western Blotting Analysis

A polyclonal antibody against OsRab7 was obtained by the injection of a purified OsRab7 peptide into rabbits. Equal amounts of protein from independent transgenic rice and wild-type rice plants were separated on a 12% SDS-PAGE gel at 4°C and then transferred onto a PVDF transfer membrane at 80 V for 2 h. The membrane was incubated in 5% w/v and nonfat milk, 0.05% v/v Tween-20, in phosphate buffered saline (PBS) for 1 h, washed three times in PBST (PBS, 0.05% Tween-20) for 10 min each, and incubated in a 1 : 1000 dilution of rabbit antiserum overnight at 4°C. After four washes with PBST, the membrane was incubated with goat anti-rabbit IgG conjugated with alkaline phosphatase (AP) in PBST solution for 2 h. After four 10 min washes in PBST, the signal was visualized by chemiluminescent detection (Pierce, Rockford, IL) according to the manufacturer's protocol.

### 2.6. Evaluation of Transgenic Rice Lines during Salt Stress

T_2_ generation seeds of* OsRab7* homozygous plants were used to assess the relative salinity tolerance. Six-day-old transgenic and wild-type seedlings grown in 1/2 MS solid medium were transferred and placed with roots pointing downward into liquid medium supplemented with 200 mM NaCl for 10 days to induce salt stress. The Data from 10 plants each of the wild-type and T1 transgenic lines were collected for each experiment, and the mean values and standard deviations were calculated and compared in phenotypic changes, the treated plants were carefully investigated and photographed. Each treatment was repeated three times.

### 2.7. Determination of Proline Content

To test the defense response triggered by *OsRab7* overexpression in transgenic rice, T_2_ homozygous plants (trans3, trans5), and wild-type plants were assayed for their proline content. Tow-week-old seedlings of both wild-type and transgenic rice were salt-treated (250 mM) for 12 h. The determination of the free proline content was performed according to Bates et al. [11]. The absorbance of chromophore containing toluene was recorded at 520 nm in a Shimadzu UV-1700 spectrophotomerter (Columbia, USA). L-proline (Sigma, USA) was used for the preparation of a standard curve.

### 2.8. Observation of Vesicle Fluorescence

Root tips from T_2_ homozygous plants and wild-type plant were, respectively, incubated with 5 *μ*g/mL FM4-64 (Sigma) for 12 min on ice. After being washed twice, the root tips were examined with a scanning confocal microscope (Olympus FV 1000). Excitation was 514 nm for the visualization of FM4-64.

## 3. Results

### 3.1. Molecular Characterization of Transgenic Rice Lines

**Figure 1:**
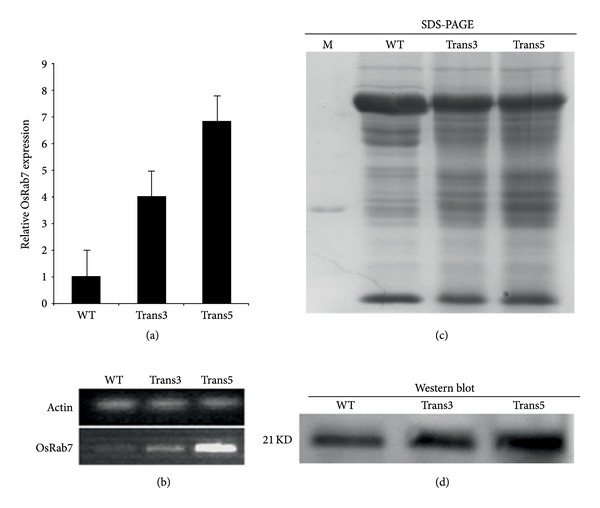
Expression of the *OsRab7* gene in transgenic rice plants. (a) Real-time quantitative PCR analysis in transgenic plants. Total RNA was extracted from transgenic plant, and wild-type plants. Actin was used as an internal control. (b) OsRab7 expression in transgenic plants was determined by semiquantitative RT-PCR analysis. (c) a SDS-PAGE showed the protein content in the samples. Equal concentrations of protein extracts from the rice samples were separated by SDS-PAGE. (d) Western blot analysis of transgenic rice plants. All lanes were run with equal amounts of the protein extracts from the rice samples.

To study the function of the *OsRab7 *gene in plants, the gene was cloned downstream of a constitutive ubiquitin promoter and introduced into rice plants via* Agrobacterium tumefaciens*-mediated transformation. Overall, 25 independent events were obtained and 23 transgenic events were confirmed by PCR firstly (Supplementary Material available online at http://dx.doi.org/10.1155/2014/483526). Analysis of the *OsRab7 *gene expression in the T_2_ progeny transgenic rice by semiquantitative RT-PCR and real-time RT-PCR showed that two homozygous lines (designated Trans3 and Trans5) exhibited a high level of transgene overexpression. As shown in Figure 1(a), the transcript levels of *OsRab7* increased to 4.2- and 7.8-fold, respectively, compared with the wild-type. Semiquantitative RT-PCR result also showed that the expression of *OsRab7* increased in Trans3 and Trans5 (Figure 1(b)). Moreover, expression of the transgene at the protein level was assayed by western blot. Equal amounts of protein from transgenic and wild-type plant were separated by SDS-PAGE gel (Figure 1(c)). An increased amount of OsRab7 protein was obviously viable in Trans3 and Trans5 compared with the wild-type plants (Figure 1(d)).

### 3.2. Evaluation of OsRab7 Transgenic Rice Plants in Salt Stress

**Figure 2:**
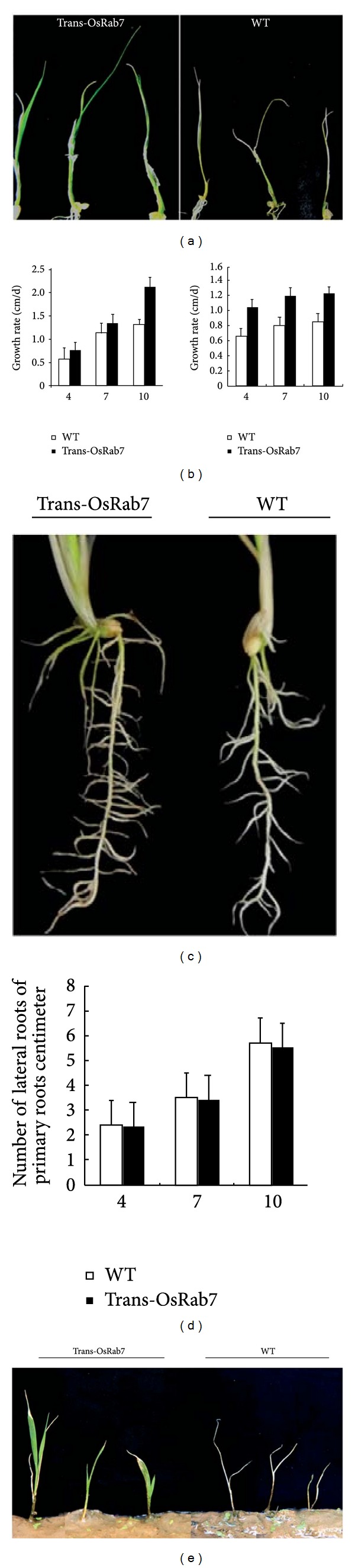
Salt stress tolerance in wild-type and transgenic plants. (a) Morphology of wild-type and transgenic seedlings after 12 days under 200 mM salt stress. (b) Growth rate compared between the transgenic rice and WT plants under salt stress. (c) Root morphology under salt stress. (d) Analysis of the number of lateral roots at 4, 7, and 10 days. The data represents the mean ± SD derived from three separate experiments. The bars represent the average data collected from 10 plants of each of the WT, Trans3, and Trans5 lines. (e) Morphology of wild-type and transgenic seedlings transplanted from salt liquid medium to soil. The wild-type and transgenic seedlings subjected to salt stress for 12 days were transplanted to soil, and the transgenic seedling growth was rescued after 8 days.

To analyze the impact of constitutive high OsRab7 expression on salt stress tolerance, the wild-type and the transgenic plants (Tans3 and Tran5) were subjected to salt stress by irrigation with 200 mM NaCl. The treatment inhibited growth in both the wild-type and transgenic plants, but the transgenic plants were much less sensitive to the treatment compared with the wild-type plants (Figure 2(a)). The seedling growth of the transgenic plant and wild-type was investigated in more detail. The seedling length of Trans5 was on average 0.18 cm, 0.22 cm, and 0.81 cm longer than that of wild-type seedlings growing at 4, 7, and 10 days, respectively, in 200 mM of NaCl. We also measured the seedling length of Trans3 at these stages and obtained the same results that the growth of *OsRab7*-overexpression transgenic rice was rapid compared with that of wild-type rice under salt stress (Figure 2(b)). Furthermore, the development of the lateral root in wild-type rice was delayed compared with that in the transgenic plants under the salt stress (Figure 2(c)). The total number of lateral roots in the transgenic seedlings was higher than that of the wild-type seedling at 4, 7, and 10 days under salt stress. However, the number of lateral roots per centimeter was almost equal to that of the wild-type (Figure 4(d)). Almost all of the wild-type plants died 12 days after the beginning of the treatment with 200 mM NaCl, whereas many of the transformed plants still had green leaves (Figure 4(a)). To address the question of whether the seedling growth would resume after removing the salt stress, the wild-type and transgenic seedlings subjected to salt stress for 12 days were transplanted to soil. As shown in Figure 2(e), transgenic seedling growth was rescued after 8 days, but the wild-type seedling growth was not recovered at the same time (Figure 2(e)). Actually, transgenic rice plants produced seeds normally 4 weeks later, whereas the wild-type almost died (data not shown). These findings demonstrated that the overexpression of *OsRab7* enhanced salt tolerance in rice.

### 3.3. The Amount of Proline Increased in *OsRab7* Transgenic Rice Plant in Salt Stress

**Figure 3:**
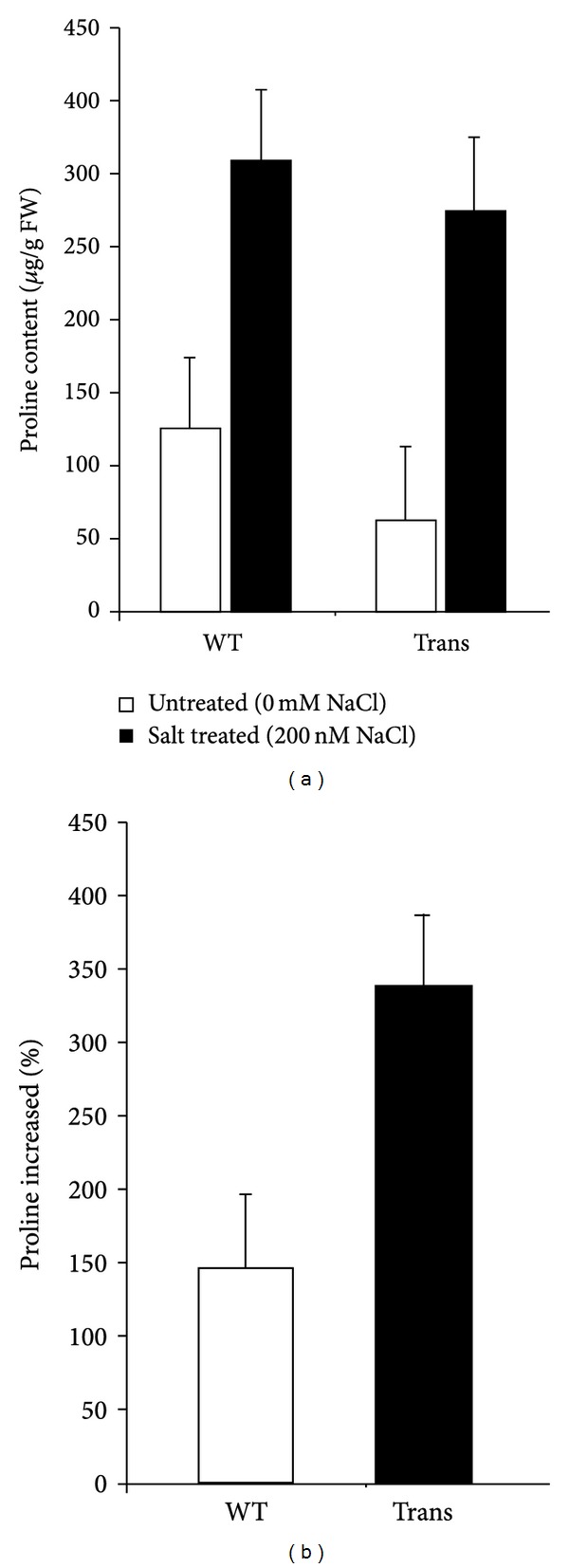
Overexpression of OsRab7 in rice increased the proline level under salt stress. (a) Level of proline in transgenic rice and WT rice under normal conditions and salt treatment. (b) Increasing rate of proline production after salt stress between the transgenic and WT rice. These data are the averages of triplicate assays and the error bars indicate the SDs.

Proline, a widely distributed osmolyte, plays an important role in enhancing abiotic stress tolerance. To investigate the physiological changes in the transgenic plants under salt stress, 2-week-old seedlings were treated with 200 mM NaCl for 24 h and then used to determine the free proline content. Under normal conditions and salt-treated conditions, increased proline levels were found in both transgenic and wild-type plants, and the proline content in the transgenic rice being slightly lower than that in the wild-type (Figure 3(a)). However, upon salt treatment, the transgenic seedlings exhibited a 3.5-fold increased proline content level compared with those under normal conditions, whereas the proline content in the wild-type plants only increased 1.5-fold after the salt treatment (Figure 3(b)). Therefore, the results indicated that the overexpression of *OsRab7* increased the proline content under salt-treated conditions, which helped transgenic seedlings to better adapt to the salt stress.

### 3.4. The Vesicles Increased in* OsRab7* Transgenic Rice Plant

**Figure 4:**
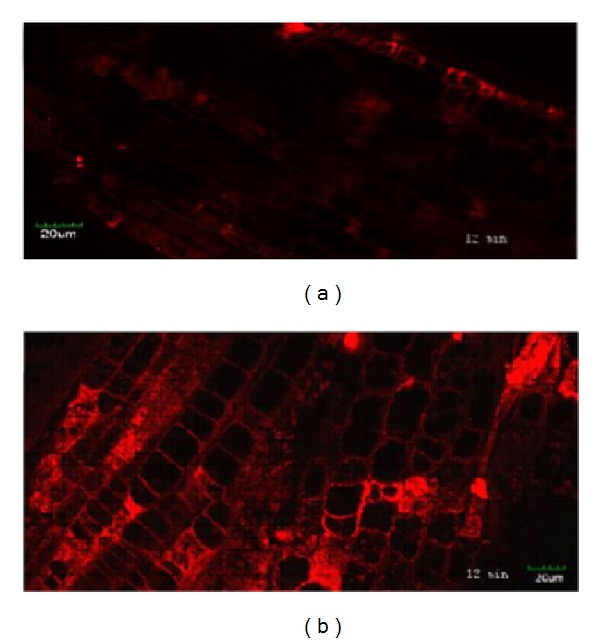
Vesicles in the transgenic plant and WT rice. (a) Vesicle morphology in the wild-type rice root tip. (b) Vesicle morphology in the transgenic plant root tip. The root tips were incubated in 5 *μ*g/mL FM4-64 (Sigma) for 12 min, and images of the vesicles were captured using confocal microscopy. Bar, 20 *μ*m.

In plants,* Rab7* plays an important role in the vesicle trafficking process, and thus, the vesicle distribution in transgenic rice was investigated using the vesicle fluorescent dye FM4-64. As shown in Figure 4, a strong, aggregated fluorescence intensity of FM4-64 was observed in the transgenic rice root tips, whereas a much weaker, diffuse fluorescence intensity was detected in the wild-type root tip. This result suggested the acceleration of vesicles trafficking in the* OsRab7* transgenic rice.

## 4. Discussion

The physiological response of plants to salt stress is a complex process requiring the coordinated function of many genes. Although* OsRab7* transcript levels were to be found increased under salt stress, direct evidence for the association of this gene with salt tolerance was lacking. In this paper, our experiments showed that the overexpression of *OsRab7* could confer salinity tolerance in transgenic rice.

Vacuoles play a critical role in salt tolerance by accumulating sodium, which is then removed from the cytosol, where it is toxic [12, 13]. A connection between salt sensitivity and vacuolar trafficking has been well established in animal cells and yeast. Post-Golgi organelles involved in vacuolar trafficking were shown to be targets of salt stress, resulting in inefficient vacuolar trafficking in yeast and mammalian cells [14–16]. In plants, Leshem et al. reported that the endocytic pathway to the vacuole was also important for salt tolerance in *Arabidopsis* [17, 18]. The Rab7 proteins are important components of the vesicle trafficking system in all eukaryotes [5]. In animals and yeast, the Rab7 protein localizes to late endosomes and to lysosomes/vacuoles [19–22]. In plants, the Rab7 proteins have been localized to the tonoplast in both *Arabidopsis* and rice [8, 23]. Moreover, enhanced vesicle trafficking and endocytic trafficking were also observed in Rab7 overexpression transgenic soybean and in animal cells containing constitutive active forms of *Rab7* [24, 25], respectively. In our studies, an increased number of distributed vesicles was observed in *OsRab7* overexpression transgenic rice, suggesting that the overexpression of OsRab7 may activate the vesicle trafficking in rice. Together with the results of salt tolerance displayed by the OsRab7 overexpression transgenic rice, we infer that the overexpression of *OsRab7* increases plant salt tolerance via enhanced vesicle trafficking in rice. Thus, our results provide additional evidence that vacuolar trafficking is important for salt tolerance.

## 5. Conclusion

In summary, we prepared transgenic rice plants that overexpressed *OsRab7 *and tested their tolerance to salt stress. The transgenic plants exhibited enhanced tolerance to salt stress and increased numerous distributed vesicles in the root tips. These results indicate that the overexpression of* OsRab7* increased plant salt tolerance via enhanced vesicle trafficking in rice.

## Supplementary Material

Additional file The OsRab7 transform cassette, and PCR confirmed the transgenic rice. (a) The *OsRab7* cassette for rice transformation with the *OsRab7* gene under the control of the ubiquitin promoter. Pubi: the maize ubiquitin promoter. (b) PCR amplification of 0.75kb of the hpt expression cassette. M DL2000 maker. 0 untransformed control rice sample; 1-23 represent the putative transformed samples.
