# Supplementary material for: Overexpression of a Vesicle Trafficking Gene, OsRab7, Enhances Salt Tolerance in Rice
Source: ScientificWorldJournal. 2014 Feb 12;2014:483526. doi: 10.1155/2014/483526 (PMC3943248; doi:10.1155/2014/483526)

Additional file The OsRab7 transform cassette, and PCR confirmed the transgenic rice.

(a) The *OsRab7* cassette for rice transformation with the *OsRab7* gene under the control of the ubiquitin promoter. Pubi: the maize ubiquitin promoter. (b) PCR amplification of 0.75kb of the hpt expression cassette. M DL2000 maker. 0 untransformed control rice sample; 1-23 represent the putative transformed samples.

a

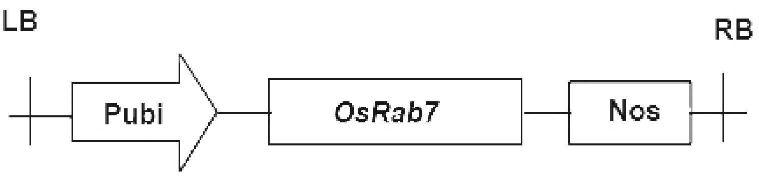

b

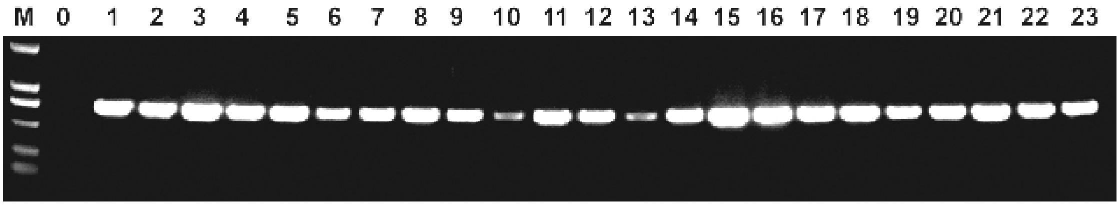

Supplement: Supplementary file 1 — Additional file The OsRab7 transform cassette, and PCR confirmed the transgenic rice. (a) The OsRab7 cassette for rice transformation with the OsRab7 gene under the control of the ubiquitin promoter. Pubi: the maize ubiquitin promoter. (b) PCR amplification of 0.75kb of the hpt expression cassette. M DL2000 maker. 0 untransformed control rice sample; 1-23 represent the putative transformed samples. [file 483526.f1.pdf]
